# Experiences and involvement of family members in transfer decisions from nursing home to hospital: a systematic review of qualitative research

**DOI:** 10.1186/s12877-019-1170-7

**Published:** 2019-06-04

**Authors:** Alexandra Pulst, Alexander Maximilian Fassmer, Guido Schmiemann

**Affiliations:** 1 Department of Health Services Research, Institute for Public Health and Nursing Research, University of Bremen, Grazer Str. 4, 28359 Bremen, Germany; 2 Health Sciences Bremen, University of Bremen, 28359 Bremen, Germany; 3 Department of Health Services Research, School VI - Medicine and Health Sciences, Carl von Ossietzky University of Oldenburg, Ammerländer Heerstraße 114-118, 26129 Oldenburg, Germany

**Keywords:** Nursing home, Hospitalization, Patient transfer, Decision making, Family, Qualitative evidence synthesis

## Abstract

**Background:**

Nursing home residents (NHR) are characterized by increasing frailty, multimorbidity and care dependency. These conditions result in frequent hospital transfers which can lead to negative effects on residents’ health status and are often avoidable. Reasons for emergency department (ED) visits or hospital admissions are complex. Prior research indicated factors influencing transfer decisions in view of nursing staff and general practitioners. The aim of this systematic review is to explore how family members experience and influence transfers from nursing home (NH) to hospital and how they are involved in the transfer decision.

**Methods:**

A systematic literature search was performed in Medline via PubMed, Ebsco Scopus and CINAHL in May 2018. Studies were eligible if they contained information a) about the decision to transfer NHR to hospital and b) the experiences or influence of family members. The review followed Joanna Briggs Institute's (JBI) approach for qualitative systematic reviews. Screening, selection and quality appraisal of studies were performed independently by two reviewers. Synthesis of qualitative data was conducted through meta-aggregation.

**Results:**

After screening of *n* = 2863 articles, in total *n* = 10 qualitative studies were included in the review. Results indicate that family members of NHR experience decision-making before hospitalization differently. They mainly reported NH-related, hospital-related, and family/resident-related factors influencing the transfer decision. The involvement of family members in the decision-making process varies - from no involvement to insistence on a decision in favor of their personal preferences. However, hospital transfer decisions and other treatment decisions (e.g. advance care planning (ACP) discussions) were commonly discussed with physicians and nurses. Conflicts between family members and healthcare providers mostly arose around the interpretation of resident’s best interest. In general, family members perceive discussions as challenging thus leading to emotional stress and discomfort.

**Conclusion:**

The influence of NHR family members concerning hospital transfer decisions varies. Family members are an important link for communication between resident and medical staff and for communication between NH and hospital. Interventions aiming to reduce hospitalization rates have to take these findings into account.

**Electronic supplementary material:**

The online version of this article (10.1186/s12877-019-1170-7) contains supplementary material, which is available to authorized users.

## Background

Nursing home residents (NHR) are a vulnerable population group with complex care needs. Many of them suffer from chronic diseases and have functional disabilities [1, 2]. Changes in resident’s health status often lead to transfers from nursing home (NH) to hospital [3]. These transfers include consecutive hospital admissions as well as outpatient treatment in the emergency department (ED). The prevalence of transfer rates varies across different countries depending on health care system and research design. According to a systematic review the incidence of transfers to EDs is at least 30 transfers per 100 residents per year [4]. A recent systematic review reported a range of hospital admissions from 6.8 to 45.7% for various time periods of follow-up [5]. The risk for hospital admissions increases in the last months of life [6–9]. These results indicate that hospital transfers are common in NHR. A high proportion of these are judged as inappropriate or avoidable [10–12]. Negative effects are in-hospital complications (e.g. pressure ulcers, nosocomial infections), functional decline, delirium and costs of increased health care utilization (e.g., for transport, examination, diagnosis and therapy) [11]. Reasons for hospital admission are often complex and multicausal. Most important factors associated with hospitalization are - for example - clinical conditions like cardiovascular events, falls and infections [3, 11] or system-related factors like staffing capacity, lack of qualification, physician’s availability or necessary equipment in the NH [13–15].

In recent years, several reviews aimed to explore factors influencing the transfer decision. These reviews analyzed the transfer process in view of nursing staff and general practitioners [13, 14, 16]. Several studies indicated that family members may play an important role in the NH, for example in the timely detection of changes in NHR’s health status [17] or acting as decision maker for the resident in case of dementia [18]. However, until now there does not exist an overview summarizing the perspectives of family members. This review aims to close this gap in the literature and summarizes family members’ experience and perceived involvement in the decision to transfer a NHR to hospital.

## Methods

The review followed the guideline of the Joanna Briggs Institute (JBI) for systematic reviews of qualitative evidence [19]. For the purpose of this review we defined a *nursing home* as a facility providing long-term nursing care for older people permanently living there. *Hospitalization* or hospital transfer (both terms are used synonymously here) was defined as a planned or an unplanned admission to hospital or ED visit. This contained also end-of-life admissions in case of an acute or palliative deterioration. With the term *family members* we defined the primary contact persons of NHR who are authorized to take decisions for them, e.g., partners, children (but also close friends or others). We used the term ‘relatives’ as a synonym.

### Eligibility criteria

We considered studies as eligible if they 1) had a qualitative or mixed-methods research design, 2) contained information about decision-making of hospitalization from NH and 3) described the experiences or involvement of family members. All publication types, except of case studies, study protocols, and editorials, were eligible for inclusion. Studies were excluded if they did not provide any information about the form of family members’ involvement (for example, quantitative studies just presenting statistical associations between hospitalizations and family members or studies which just described the presence of available relatives). Studies were also excluded if they were related to other care settings (short-term care, assisted living facilities and home care).

In the full text screening we included studies which directly included family members as study participants. Because we intended to investigate the decision-making process in the NH *before* hospitalization, results related to the situation after returning back to the NH were not included.

### Literature search

The Cochrane Handbook recommends three bibliographic databases as the most important sources to search for potential studies (CENTRAL, MEDLINE, EMBASE) in systematic reviews [20]. Because CENTRAL focuses on trials which were not eligible for our review question, we chose CINAHL as nursing-related database instead. Searching in SCOPUS included most EMBASE content. We conducted systematic literature in Medline via PubMed, Cumulative Index to Nursing and Allied Health Literature (CINAHL) and Ebsco Scopus on 30 May 2018.

Based on objective of the review and the **PiCo** template for qualitative reviews [21], the search combined sets of terms for **P**opulation (*family members*), the phenomenon of **I**nterest (*hospitalization*) and the **Co**ntext (*nursing home*) using MeSH terms (Medical Subject Headings) and text words (see Additional file 1 for literature search strategy). Keywords for the search strategy were derived from an initial limited search in Medline via PubMed. In addition, the search strategy of a thematically similar published review [5] was used and complemented for the purpose of this article. Manual search was performed on reference lists of articles for additional material. Further, we used Google Scholar to identify grey literature by combining the terms “nursing home”, “hospital”, “transfer/admission” and “family/relatives”. There was no limitation in time period to identify all relevant literature. Language was no exclusion criteria.

### Quality appraisal

The methodological quality of the included studies was assessed independently by two reviewers (AP, AF) using the JBI Critical Appraisal Checklist for Qualitative Research [22]. Disagreements between reviewers were solved by discussion and if no consensus could be found, results were discussed after independent assessment of a third reviewer (GS). Results of quality assessment are shown in Additional file 2.

### Data extraction

Data relevant to the review question were extracted from the included studies using an adapted version of the data extraction tool from JBI Qualitative Assessment and Review Instrument (JBI-QARI) [19]. Following information were extracted: authors, year, country, study focus/phenomen of interest, method of data collection, method of data analysis, participants, setting and key findings (Table 1). The first reviewer (AP) conducted data extraction for each study and was checked by a second reviewer (AF).

**Table 1:** Characteristics of included studies

Authors (year), country	Study focus/phenomenon of interest	Method of data collection	Method of data analysis	Participants	Setting	Key findings
Abrahamson et al. (2016) USA [23]	Experiences of family members in the NH to hospital transfer decision-making process.	Semi-structured interviews (telephone)	Qualitative content analysis	Family members(*n* = 20) involved in transfer decision of residents with dementia within the past three months	9 NHs	Even though family members appreciated staff’s good knowlegde of the resident, they perceived NHs as providing” low-level “medical care. Hospitalization decisions were influenced by family members’ perception that physicians’ presence in NH was lacking, nursing staff was unable to notice changes quickly enough and that more care was available in hospital.
Arendts et al. (2015) Australia [29]	Perspectives of residents, relatives and nursing staff concerning transfers from NH to ED and influencing factors.	Semi-structured interviews (face-to-face)	Qualitative content analysis	Residents (*n* = 11) with previously ED admission without life- or limb-threatening reason for transfer and cognitively able to participate Family members (*n* = 14) Nursing staff (*n* = 17), (nurses or nursing assistants)	6 NHs	Depending on the relationship to nursing staff, family members’ involvement in transfer decisions differed. They mostly accepted staff’s advice with or without communication of the situation. Because of negative impacts on their relative, family members often tended to avoid transfer – but recognizing that transfer was often necessary. They perceived that transfers were motivated by lack of nursing staff and physicians in NH or risk-averse behaviour of nursing staff.
Arendts et al. (2010) Australia [30]	Factors influencing transfers decisions from NH to ED and interventions that could reduce number of transfers.	A) Three focus groups: 1) Family members, community representatives, non-health professional carers 2 + 3) family members, NH staff, ED staff, GP B) Semi-structured interviews	Thematic analysis	A) In total (*n* = 33): family members (n = 5) NH carer (*n* = 7) NH nurses (*n* = 5) NH manager (n = 5) ED staff (*n* = 7) GP (*n* = 4) B) Residents (*n* = 9) previously been transferred to ED	Not stated	The perceptions of family members and nursing staff corresponded concerning following aspects: understaffing, insufficient qualification of staff (e.g. end-of-life) and lack of physicians providing care in NHs. In addition, nursing staff perceived bureaucracy of GP visits and insufficient communication with ED staff as difficult providing adequate care. Interventions that could reduce transfers aimed on training of NH staff (clinical procedures, palliative care) and families (information about end-of-life care) as well as new organizational structures.
Carusone et al. (2006) Canada [27]	Resident and family members’ perspectives on in situ care for pneumonia in NHs	Semi-structured interviews (face-to-face + telephone)	Thematic analysis	Residents (*n* = 6) with recent case of pneumonia and capable of making their own decisions Family members (*n* = 8) directly involved in decision-making for residents unable to speak about their own care	4 NHs	Family members took pneumonia as a condition manageable in NH. They generally preferred care in NH because of more personal attention and comfort. On the other hand hospital-based care seemed to be more adequate because of understaffing, lack of physicians and lack of necessary equipment in NHs. In contrast to residents which ceded the transfer decisions to nursing staff or the physician, family members wanted to be involved in decision-making and were mostly thankful for staffs’ recommendations.
Dreyer et al. (2009) Norway [31]	Relatives’ experiences of decision-making processes when life-prolonging treatment of residents is limited.	(Semi-structured) in-depth interviews	Constant comparative analysis	Family members (*n* = 15) (children, spouse, children-in-law)	10 NHs	Relatives were mostly not contacted until resident’s condition deteriorated. The knowledge about residents’ wishes and recognition of end-of-life symptoms was insufficient among family members. Involvement in end-of-life decision was therefore associated with emotional burden. Hospitalization was not discussed with relatives.
Kayser-Jones et al. (1989) USA [24]	Clinical conditions and social-cultural factors contributing to hospitalization of NH residents	Event analysis of 215 acute-illness episodes Semi-structured interviews	‘Qualitative analysis’	Residents (*n* = 215) Family members (*n* = not stated)	3 NHs	Frustration and fear about conditions of NH care (e.g. inadequate nursing skills) triggered relatives to insist on hospitalization. On the other hand hospital care was sometimes described as traumatic experience in an unfamiliar situation. Hospitalization was therefore mostly influenced by social-structural rather than clinical reasons.
Robinson et al. (2012) Canada [28]	Key elements influencing the success of transitions of residents moving between NHs and EDs in the perspective of residents, family members and health care providers within three settings	A) Semi-structured interviews (*n* = 24) (individual and group interviews) B) Focus groups (*n* = 6) C) Interviews (*n* = 7)	Constant comparison analysis	A) Residents (*n* = 7) with previous transition from NH to ED within the last 12 months + family members (*n* = 20) B) Healthcare provider (*n* = 37), registered nurses, licensed practical nurses, paramedics, physicians, and administrators C) Healthcare provider (*n* = 7)	NHs EMS ED	Family members are mostly seen as key figures in decision-making process because of knowing the resident. They were able to fill in information gaps between different healthcare providers. However, family members also felt uncomfortable in case of ACP discussions. Conflicts with nursing staff typically occurred around the interpretation of resident’s best interest and because of different perspectives.
Tappen et al. (2016) USA [25]	Residents and family members’ perspectives on decision-making process when confronted with a (real or hypothetical) possibility of transfer to acute care when a change in the resident’s condition occurred.	Integrated mixed methods design 171 semi-structured interviews based on Critical Decision Method	‘Qualitative analysis’ + Quantitative analysis of sociodemographic data compared to decision mode	Residents (*n* = 96) Family members (*n* = 75) (children (*n* = 36), spouses (*n* = 26), others (i.e. sibling, grandchild, parent) (*n* = 13)	19 NHs	Decision-making was driven by different aspects among family members. Weighing pros and cons of hospital-based and NH-based care was the most common behaviour. In a few cases hospital decisions were emotion-based because of relatives’ positive or negative experiences about care in NH or hospital in the past. Every fifth family member completely trusted others and delegated the decision to nursing staff or physician.
van Soest-Poortvliet et al. (2015) Netherlands [32]	Experiences with the process of ACP in NH residents with dementia. Factors related to the timing and content of ACP as perceived by family, physicians and nurses.	In-depth interviews (*n* = 65) (Telephone + face-to-face)	Thematic analysis	Family members (*n* = 20) Physicians (*n* = 21) Nurses (*n* = 24) Perspectives of all 3 caregivers available on 14 of the 26 patients	16 NHs	Care goals, treatment decisions (e.g. cardiopulmonary resuscitation) and hospitalization were commonly discussed together with physicians, nursing staff and family members. Hospital transfers were mostly initiated by the physician when change in resident’s health status occurred. Sometimes family members’ willingness to ACP discussions was limited.
Waldrop et al. (2011) USA [26]	Family members’ experiences with a dying NH resident and the living–dying interval in a NH	Interviews In-depth interviews	‘Qualitative data analysis’	Family members (*n* = 31) of 27 NH residents who had died 2 months previously	1 NH,	Family members favoured hospital transfers in cases of sudden deterioration. In some instances they were not aware of typical end-of-life symptoms or ignored the imminent death of their relative. Being confronted with end-of-life situation caused stress among family members which sometimes led to conflicts between them and nursing staff.

*NH* nursing home, *ED* emergency department, *GP* general practitioner, *EMS* Emergency Medical Services, *ACP* advance care planning

### Data synthesis

For managing data synthesis we used the software MAXQDA Analytics Pro 2018. Articles were analyzed independently by two persons to develop a list of thematic codes and subcodes (open coding). The codes were not specified prior to analysis and therefore derived from the text solely. Discrepancies between reviewers were discussed until consensus led to a final code list. The articles were then re-analyzed using this list.

Based on the JBI guide for data synthesis [19] meta-aggregation was used to synthesize qualitative data. The following three steps were conducted: 1) all text passages and quotes relevant to the review question were extracted from the results, discussion and conclusions of each study (also in the abstract). The illustrations were synthesized to findings which were rated according to JBI-QARI levels of credibility (U=Unequivocal; C=Credible; NS=Not supported). 2) Findings were summarized to categories and subcategories based on similarity in meaning. 3) Synthesized findings were derived from the categories. Data synthesis was performed by the first reviewer (AP). As validation, the analysis of findings was discussed with the other authors.

## Results

### Screening and search outcome

**Fig. 1:**
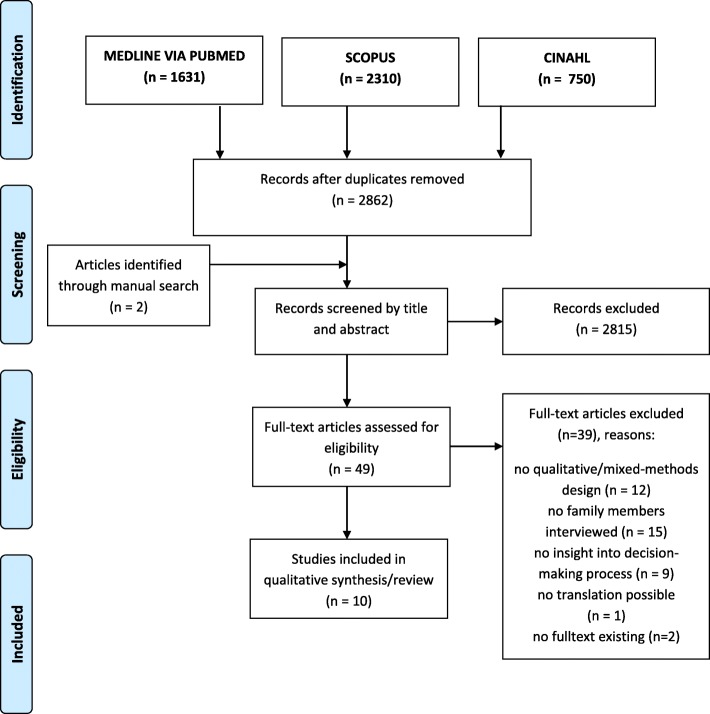
Study selection and screening process

The primary literature search identified 4691 articles. Additionally, *n* = 2 articles were found through manual search. After removing duplicates two researchers (AP, AF) independently screened title and abstract of *n* = 2862 articles using the software tool *Rayyan* [23]*.* Any potentially relevant publication (*n* = 49) was ordered in full-text and assessed for inclusion and exclusion according to eligibility criteria, following the same procedure. After full-text screening *n* = 39 studies were excluded, one of them because translation of a Japanese paper was not possible (see Additional file 3 for list of excluded studies). Any disagreement in the process of selection and assessment was solved by discussion and if necessary by a third researcher (GS). In total *n*=10 studies were included in this systematic review (Fig. 1).

### Description of included studies

The included studies (Table 1) were published between 1989 and 2016, most of them after 2010. Four studies were conducted in the USA [24–27], two each in Canada [28, 29] and Australia [30, 31], and one each in Norway [32] and the Netherlands [33]. Eight studies used semi-structured interviews, in-depth interviews or focus groups.

Two studies conducted a mixed-methods design combining participant observation and interviews [25] or combining interviews and quantitative analysis [26]. Eight studies used qualitative design. Data were analyzed using thematic, content or comparative analysis or is just described as “qualitative analysis” [25]. The studies focused on the family members’ perspective on acute changes in residents’ health status, the decision-making process around hospital transfers, influencing factors and dealing with end-of-life care, death or limited prolonging treatment. Referring to the inclusion criteria, participants of the studies were family members only [24, 27, 32] or a combination of family members and residents, nurses, physicians or other healthcare providers (*n* = 8) [25, 26, 28–31, 33]. Referring to our broad definition of “family members”, none of the studies included perspectives of close friends, neighbors or others.

### Quality of studies

Using JBI-QARI tool the critical appraisal showed variation in the quality of the 10 included studies. The percentage of quality criteria answered with ‘yes’ varied between 4 of 10 (40%) and 9 of 10 (90%) in each study (see Additional file 2). Referring to the objectives all studies used appropriate methods to answer their research question. Most studies represented participants’ voices adequately through quotations, except one [25]. However, most of the studies did not provide a statement locating the researcher culturally or theoretically. The influence of the researcher on the research was addressed in none of the studies. One study did not provide information about ethical approval [25]. In conclusion the quality of the included studies can be rated as moderate to high.

### Findings and categories

**Fig. 2:**
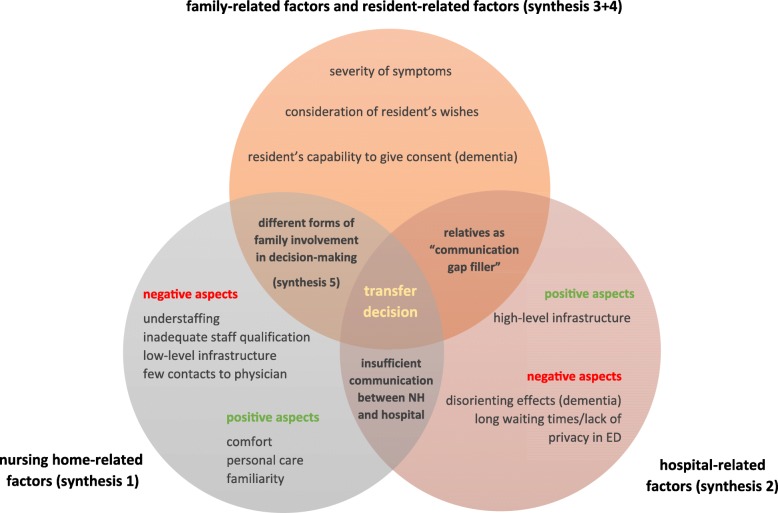
Factors influencing hospital transfer decision - overview of family members’ experiences

Seventy-four illustrations were extracted from the 10 included studies. These were analyzed and grouped into 18 categories based on similarity in meaning. The categories were clustered into 5 synthesized findings: ‘nursing home-related factors (synthesis 1), ‘hospital-related factors (synthesis 2), ‘family-related and resident-related factors’ (synthesis 3 + 4) and ‘forms of family involvement’ (synthesis 5) (Fig. 2).

### Synthesized findings

#### Synthesis 1: transfer decision is affected by family members’ judgement of quality of NH care (nursing-home related factors)

This synthesized finding emerged from two categories: positive NH experiences and negative NH experiences. Depending on how family members perceive daily care in the NH or which expectation they have, their tendency to hospitalize their older relative can differ. On the one hand family members favored the *“personal care”* [28] in the NH and associated it with *“familiarity”* [24] and *“comfort”* [24, 28] which had a positive impact on “*quality of life”* [24]. Robinson et al. (2012), for example, concluded in their study: *“When family members observed healthcare providers treating their relatives with compassion, dignity and respect, their relationships with healthcare providers were supported by trust, confidence and admiration”* [29]. These factors seem to be beneficial for remaining in the NH and **avoiding hospitalization**.

On the other hand studies described several negative experiences in the NH: family members argued that especially understaffing [28, 30, 31] is problematic when NH staff is not able to react quickly enough to resident’s changes in health status [24]. Also the qualification of NH staff [25, 31] is a main concern when a change in resident’s health status occurs. In this context, family members sometimes observed *“risk-averse”* behavior when nursing staff *“has to cover themselves. Overreaction is probably the wrong word but they’re overcautious”* [30]. Kayser-Jones et al. (1989) even concluded that *“families reported feeling frustrated by what they identified as inadequate nursing skills and, fearful when their relative’s condition worsened, sometimes urged the physician to transfer him/her to an acute hospital”* [25]. Besides that, family members criticized the lack of physician’s availability, [24, 30, 31] and necessary equipment in the NH [28]. In summary, these impressions led to the opinion that NH just can provide low-level care which increases the **risk of hospitalization** [24].

#### Synthesis 2: transfer decision is affected by family members’ judgement on quality of hospital care (hospital related factors)

Family members who had negative experiences in the NH, concurrently talked about their positive attitude towards hospital treatment. *“If we hadn’t had a good hospital experience, we might have been more influenced to stay here* [comment of the authors: in the NH]” [26]. The most frequently mentioned **benefits of hospital care** were available medical equipment and infrastructure [24, 28]. One family member who decided about place of care for his relative with pneumonia pointed out: *“[In the hospital] it’s not a question of taking blood, sending it to the laboratory and having somebody come back three days later... they immediately check it and they know exactly [what is going on*] ”[28]. Other family members also advocated *“more care around the clock”* in the hospital [26].

Particularly in the case of dementia hospital treatment can have a *“disorienting effect”* [30], can cause *“confusion”* [28] because of *“lack of attention to the needs of aged patients with cognitive or functional impairments*” [24]. In these cases family members tended more to **avoid hospitalization**. *‘To transfer [my mother] to hospital and get use to the hospital environment, I think is more detrimental...[even though] I think they would get more superior treatment in the hospital... and the medical staff assessment there would be far superior than in the nursing home’* [28]. But also regardless of a dementia diagnosis, some family members described hospital transfers as a “*trauma experience […] without adequate explanation, to an unfamiliar location, with unfamiliar staff and an unfamiliar physician”* [25]. Especially in the ED *“waiting times”* and *“lack of privacy and cool ambient temperature”* [30] led to discomfort influencing family members’ attitude towards hospitalization.

#### Synthesis 3: perceived severity of clinical situation effects the transfer decision (family-related and resident-related factors)

The tendency to transfer the relative to hospital depended also on **severity of symptoms**. Family members favored hospital transfers when *“a dramatic change in the person’s condition”* occurred [27]. *“Hospital care is clearly necessary for some conditions (e.g. fainting, broken bones, operations, and heart problems)”* but for example, *“not for pneumonia”* [28]. In this context one study indicated that if the reason for transfer was viewed as *“non-life threatening”,* advance directives were not considered and seen by the family as not *“applicable to the situation”* [24]. But severity of symptoms is not always clear. Kayser-Jones et al. (1989) argued: *“Transfers also occurred when there was uncertainty about the severity of the patient’s condition. In such cases, especially if a concerned family member was present, physicians hospitalized the patient out of indecision and/or fear of litigation”* [25].

#### Synthesis 4: knowing, accepting and upholding resident wishes are challenges for family members (family-related and resident-related factors)

In the included studies family members dealt differently with resident’s preferences. Some of them knew the wishes of their older relative, others did not. If the residents’ **wishes were unknown**, this is because talking about death *“was a sort of****taboo****”* within the family [32]. Often transfer or treatment decisions were discussed with family members [24, 27, 29, 30], but actually they felt uncomfortable in their role as decision makers. Van Soest-Poortvliet et al. (2015) for example showed that **willingness to discuss end of life situations can be limited** and summarized: *“Some families are open for discussions about the end of life and it is not difficult to make decisions. However, other families have to get used to the NH. They absolutely did not want discussions about end of life immediately*” [33]. In single cases there may be relatives who either do not **recognize end of life-symptoms** (e.g. resident stops drinking) [32] or **suppressed the fact** that their loved one is **approaching death** [27]. *The nurse said, “Maybe she should be under hospice care.” I said, “Oh definitely. No problem with hospice.” But that was like a lightning bolt and the first time I really thought, “Oh my gosh, she’s dying”* [27].

If resident’s wishes were known, relatives reported that **upholding these wishes** can be very challenging, for example if relatives did not agree with them [24, 27, 30]. On the other hand, Dreyer et al. (2009) described the tendency of some family members to **override resident’s wishes**. This may happen when residents – independent from being able to give consent or not – refused to be fed. As a consequence, family members forced their loved one to eat or to drink. The authors pointed out in this context: *[…] not all the relatives acted in the patient’s best interests. […] personal preferences, feelings and viewpoints could dominate. Some wanted life-prolonging treatment because they were afraid of the loss they would experience. Others had lost one of their parents earlier, had not done enough for the dying parent then, and wanted to do “everything possible so that they would not be left with a bad conscience again”* [32].

#### Synthesis 5: the extent of family members’ involvement in treatment and transfer decisions vary (forms of family involvement)

In daily care of NHs treatment decisions and hospital transfer decisions are often required. Family members are differently involved in this decision-making process – from no involvement to insistence of several family members’ to decide in favor of their personal preferences. Dreyer et al. (2006), for example, described that relatives were often **not contacted** until the health status of their loved ones deteriorates. Especially in case of dementia and when acute events occurred (e.g. suspected stroke) physicians and nursing staff decided about hospital transfers without discussion with the family [32].

However, in daily care family members have most contacts with nurses. Some relatives reported that they completely trust in the physician’s and staff’s medical know-how and therefore **ceded/delegated decisions** to them [26, 30] - *“I wouldn’t decide anything. I would talk to the doctor. To tell you the truth, I would tell them, if they feel that they can do it here that is alright or either carry her to the hospital. It’s up to them. I wouldn’t try to boss them too much”* [26]. Despite the reporting of family members that a contact with physicians in the NH were often missing [24, 30], several studies showed that physicians and nursing staff discussed treatment or hospital transfer decisions **together** with the resident and the family if that was desired [28–30, 32, 33]. In case of an acute and *“urgent”* situation [29] it was also considered acceptable to inform the relatives immediately after hospital transition. Also advance care planning (ACP) discussions commonly took place prior to the hospitalization [24]. Van Soest-Poortvliet et al. (2015) reported that discussions about hospitalization and resuscitation mostly took place directly or soon after admission. They were often initiated by the physician and resulted in do-not-resuscitate (DNR) and a do-not-hospitalize (DNH) orders. During these discussions some family members stated to feel *“uncomfortable”* [29] in their role, especially when they had a lack of medical knowledge [29]. Waldrop et al. (2011) suggested: *“decisions that occurred in the heat of the moment were painful and difficult for family members”* [27]. Therefore, most relatives were usually thankful for **recommendations and took staffs’ advice** [28–30]. *“They felt they needed to call the ambulance and get her back there. And they said how do you feel about that? Can we call the ambulance and get her back there? And I said if you feel she needs the ambulance – needs hospital – to get back there, please do [relative 11]”* [30]. In this context, Robinson et al. (2012) pointed out that involvement of the family is highly influenced by the relationship between the family and the NH staff/physicians. On the one hand, family members were valued as important and supportive key members in decision-making [29]. In the transition process information about the resident and her/his medication sometimes got lost [24]. Family members were able to *“fill in the gaps”* [29] in the communication between the NH and the hospital: *“For example, family members were critical to helping ED providers ‘know’ the resident and sometimes provided the only report to NH staff about what happened in the ED”* [29].

On the other hand, in some cases the decision making-process of hospital transfers or ACP discussions can cause **conflicts** between relatives and NH staff [25, 27, 29, 32]. Tensions *occurred “typically around interpretation of the resident’s best interests and discrepancies in perspective”* [29]. Such conflicts appeared when family members disagreed with the physician’s recommendation of a transfer because their loved one did not want to go to the hospital. On the contrary, when nursing staff believed that “*it was in the resident’s best interest to remain ‘at home’*, especially family members “*at a geographic distance [...] wanted ‘everything done’” for their relative”* [29]. Conflicts can also arise when family members felt *“frustrated by what they identified as inadequate nursing skills”* and were *“fearful when their relative’s condition worsened”* [25]. In all these cases **pressure/insistence** from the family can influence hospital transfers or ACP decisions.

## Discussion

Many studies examined influencing factors on transfer decisions from NH to hospital. Most of them reported the decision-making process in the perspective of nursing staff and physicians. This review aimed to extend the existing evidence by analysing experiences and involvement of family members when a hospital transfer decision occurs. Because of thematic similarity the studies did not only deal with hospital transfer decisions, but also with end-of-life decisions like limited prolonging treatment and ACP.

Being confronted with treatment or transfer decisions family members often reported a lot of discomfort and emotional stress. Even though residents reported to trust relatives making decisions for them [34], relatives themselves felt insecure in these situations. Especially if the older residents are unable to give consent (e.g. in case of dementia), if their wishes are unknown or do not correspond with relatives’ preferences, these situations are perceived as very challenging. There can be reasons to believe that decisions made by family members tend to represent more their own wishes rather than the preferences of the resident [35]. Therefore, some situations might cause **conflicts** between relatives and nursing staff in the discussion of resident’s best interests. The existence of advance directives might not be sufficient to solve this problem because 1) just few residents possess advances directives [36] and often they are incomplete [37] and 2) therefore many residents were still transferred to hospital despite of having a DNH-order [38, 39]. The results of our review indicate that interventions trying to prevent hospital admissions should take into account the influence of relatives. As Dreyer et al. (2009) reported, relatives **fear death/losing a loved one or live with bad conscience**. Cohen et al. (2017) described similar aspects when guilt pushes families to *“do everything”* which includes hospitalization: *“Essentially people will say that you’re giving up. ‘You mean you didn’t send her this time? You gave up’”* [40]. Physicians just rarely discuss psychological (e.g. sadness and fear of death), spiritual or existential problems (e.g. difficulty in accepting the situation) with residents and relatives during the last months before death [41]. Intensive discussions at NH admission about treatment preferences, concerns amd regular support of a social worker/pastor/chaplain might be helpful responding to relatives’ needs of communication and information. Further research is needed analysing if such interventions may have an impact on hospital transfer rates.

Besides that, we found that the attitude of family members towards hospital transfers mainly depends on their **individual positive and negative experiences** regarding NH and hospital care. If personal care is desired, relatives assume that the NH is the more suitable setting for further treatment. On the other hand, family members tend to accept hospitalization if they associate 1) hospital care with quick medical examination and high-level infrastructure and 2) NH care with understaffing, insufficient staff qualification or lack of physician’s availability. These aspects corresponded with reporting of nursing staff in other studies [14, 15, 42–44]. In addition, general practitioners stated that clinical picture, medico-legal issues, workload [45] and communication between healthcare professionals increase the tendency for hospitalization [46]. Comparing relatives’ experiences to the statements of medical staff, it seems to be that both perceive the same problems when talking about hospital transfer decisions.

Family members described their **extent of involvement** in decision making very differently. A study of Petriwskyj et al. (2014) explored family involvement in decision-making explicitly focusing on residents with dementia – the results mainly correspond with our review showing that participation of relatives varied from total control to delegating the decision to medical staff [18]. Across the included studies in this review, relatives reported to discuss treatment and transfer decision with the physician (and sometimes the resident). Family members argued not being able to assess residents’ complaints - and therefore relying completely on the expertise of medical staff taking their advice/treatment recommendations. This was also shown by a study in Norway [47]. Nevertheless, nursing staff considered family members playing a key role because they often act as “gap filler” between NH and hospital when information gets lost during transfer. Manias et al. (2015) described in this context that family members are, for example, able to solve medication-related problems in the hospital when previously being involved in medication activities at home [48]. Relatives can also be an important link between the resident and the nursing staff - for example by noticing timely signs of changes in health status and informing or educating nursing staff about these changes [17].

### Strengths and limitations of the review

To the best of our knowledge this is the first systematic review which focuses on the experiences of family members and gives an overview of their involvement in the decision-making process of hospitalization. Just three of the ten studies focused on family members solely [24, 27, 32]. The other studies interviewed also residents and/or other healthcare providers and summarized their findings across all participants. The extraction of family members’ perspectives was therefore less accurate which might be a limitation. To minimize the risk of biased results and overlooking relevant text passages, data extraction was conducted independently by two reviewers and extended to the results, discussion and abstract for each study. Because data analysis was conducted based on published research articles (without the original transcripts), coding of the text passages was mainly dependent on the quality of the codings in the original articles.

Most of the included studies had relevant methodological shortcomings, for example the study by Kayser-Jones et al. (1989) [25] reached in the assessment only a score of 40%. However, the contained information are concordant to the results of other included studies. The cultural and theoretical background of the researcher might influence the results of the studies. The included studies neither provided information on the researchers’ background nor discussed these aspects further. As scientific backgrounds of the authors differ, we assume that these limitations are unlikely to influence the results of our review.

The effects of potentially influencing factors like NH ownership or health system-related characteristics were not described in the included studies. In addition, majority of the studies were conducted in the USA, Canada and Australia. Therefore, the results are limited to Western countries and especially to Northern America and Australia.

## Conclusion

The results of this review show that relatives’ perceptions of transfer and treatment decisions are mainly influenced by positive and negative care experiences in the NH and hospital, individual preferences and the relationship between nurses, relatives and physicians. Involvement of family members in decision-making varies from no involvement to total control about decisions. Generally, being confronted with hospitalization decisions and end-of-life issues is very stressful and challenging for relatives. Nevertheless, family members are an important link between resident and medical staff as well as between NH and hospital. These insights should be taken into account when developing interventions to reduce hospital transfers from NH. Further research, especially in European countries is needed to examine generalizability of the results on other populations.

## Additional files

Additional file 1: Literature search strategy. (XLSX 12 kb)

Additional file 2: Results of JBI critical appraisal. (XLSX 13 kb)

Additional file 3: List of excluded studies. (XLSX 9634 kb)

## Data Availability

The datasets used and analysed during the current study are available from the corresponding author on reasonable request.

## Abbreviations

ACP: Advance care planning
CINAHL: Cumulative Index to Nursing and Allied Health Literature
DNH order: Do-not-hospitalize order
DNR order: Do-not-resuscitate order
ED: Emergency department
JBI: Joanna Briggs Institute
JBI-QARI: JBI Qualitative Assessment and Review Instrument
MeSH: Medical Subject Headings
NH: Nursing home
NRH: Nursing home residents
